# Variations in vertical mucosal thickness at edentulous ridge according to site and gender measured by cone-beam computed tomography

**DOI:** 10.1186/s40729-021-00319-w

**Published:** 2021-05-12

**Authors:** Motohiro Munakata, Koudai Nagata, Minoru Sanda, Ryota Kawamata, Daisuke Sato, Kikue Yamaguchi

**Affiliations:** 1 Department of Implant Dentistry, School of Dentistry, Showa University, 2-1-1, Kita-Senzoku, Ota-ku, Tokyo, 145-8515 Japan; 2 Department of Oral and Maxillofacial Implantology, Kanagawa Dental University, Yokosuka, Japan; 3 Department of Prosthodontics, School of Dentistry, Showa University, Tokyo, Japan

**Keywords:** Mucosal thickness, Dental implant, Cone-beam computed tomography, Peri-implant mucosa, Vertical mucosal thickness

## Abstract

**Background:**

The vertical thickness of the peri-implant mucosa is associated with the amount of post treatment marginal bone loss. However, the variations in mucosal thickness at the different edentulous sites have been sparsely documented. The purpose of the study was to conduct a survey of the frequency distribution of variations in mucosal thickness at the different sites of the edentulous alveolar ridge and to compare them according to gender.

Our study included 125 partially edentulous patients having a total of 296 implant sites. Cone-beam computed tomography (CBCT) scans were obtained by placing a diagnostic template with a radiopaque crown indicator on the ridge to determine the mucosal thickness at the crest of the alveolar ridge.

**Results:**

The mucosal thickness was 3.0±1.3 mm in the maxilla, which was significantly greater than the mucosal thickness of 2.0±1.0 mm in the mandible (*p*<0.001). In both the maxilla and the mandible, the mucosa was the thickest in the anterior region, followed by the premolar and molar regions. Sites were further classified into two groups based on whether the mucosal thickness was greater than 2 mm. In the mandible, more than half of the sites showed a mucosal thickness of 2 mm or less.

**Conclusions:**

Although this study was a limited preoperative study, the vertical mucosal thickness at the edentulous ridge differed between the maxillary and mandibular regions. The majority of sites in the mandibular molar region had a mucosal thickness of less than 2 mm. Practitioners might be able to develop an optimal dental implant treatment plan for long-term biologic and esthetic stability by considering these factors.

## Background

Since the beginning of implant dentistry, the preoperative evaluation of hard tissues, especially the alveolar bone, has been a major concern of surgeons in the accurate planning and execution of implant placement. Therefore, much has been studied and published regarding the radiographic evaluation of the alveolar ridges, including the anatomical variations, evaluation methods, and bone quality classifications.

However, the significance of soft tissue in implant success was recognized later than that of hard tissue. Berglundh et al. [1] investigated the so-called “biological width” and concluded that a certain distance was required to form a soft-tissue seal to defend against infection, similar to the periodontal tissue in peri-implant mucosa. It has also been reported that placing an implant at a site having thin mucosa leads to bone resorption, which is considered to be for the acquisition of biological width [1]. Vervaeke et al. [2] reported that placement of the final restoration also causes significant bone resorption in cases with thin peri-implant mucosa. Moreover, systematic reviews have reported intensified crestal bone loss in cases with 2 mm or thinner peri-implant mucosa compared to the cases with thicker peri-implant mucosa, which implies the significance of biological width [3, 4]. Therefore, evaluation of the mucosal thickness at the peri-implant site [2] can significantly influence the depth of insertion in consideration to the biological width, biotype, esthetics, biological tissues related to mucosal changes and bone resorption, and anatomical conditions of the peri-implant mucosa [5].

Several methods for evaluating soft tissue have been investigated; however, there are few established methods for the preoperative evaluation of soft tissue. For instance, it has been proven that measuring mucosal thickness using anesthetizing needles generally lacks objectivity [6, 7]. Furthermore, although several studies have been conducted in recent years on the effect of mucosal thickness on crestal bone loss, most studies have confined their investigation to mandibular molar sites, and tissue thickness has been measured during surgery [8–13]. Non-surgical and pre-surgical measurements of mucosal thickness at the edentulous ridge have not been attempted much. Besides, the frequency distribution of mucosal thickness at the various edentulous sites, which would certainly influence the preoperative decision-making process, has not yet been elucidated.

In this study, we used cone beam computed tomography (CBCT) to evaluate the variations in mucosal thickness at the crest of the alveolar ridge where implants were to be placed and compared the obtained data based on gender and the location of edentulous sites.

## Methods

### Subjects

Partially edentulous patients who received dental implant treatment at Kanagawa Dental University Hospital and Showa University Dental Hospital between September 2015 and November 2018 were included in the study.

The inclusion criteria were as follows: age >18 years, good general health, bone crest with a minimum width of 6 mm, presence of keratinized mucosa with a minimum buccolingual width of 3 mm, no bone augmentation procedures before implant placement, willingness of patient to undergo CBCT, capability to fully comply with the study protocol, and willingness to give written informed consent. The exclusion criteria were as follows: CBCT without a radiographic stent, severe periodontitis, CBCT within 3 months after tooth extraction, cases without corticalization of the alveolar ridge, cases with CBCT images showing air transmission at the bottom of the template, metabolic bone disease or syndrome, such as osteoporosis and rheumatoid arthritis, history of radiation therapy in the head and neck region, heavy smoking (>10 cigarettes/day), history of bisphosphonate administration, and uncontrolled diabetes.

### Instrumentation/measurements

**Fig. 1:**
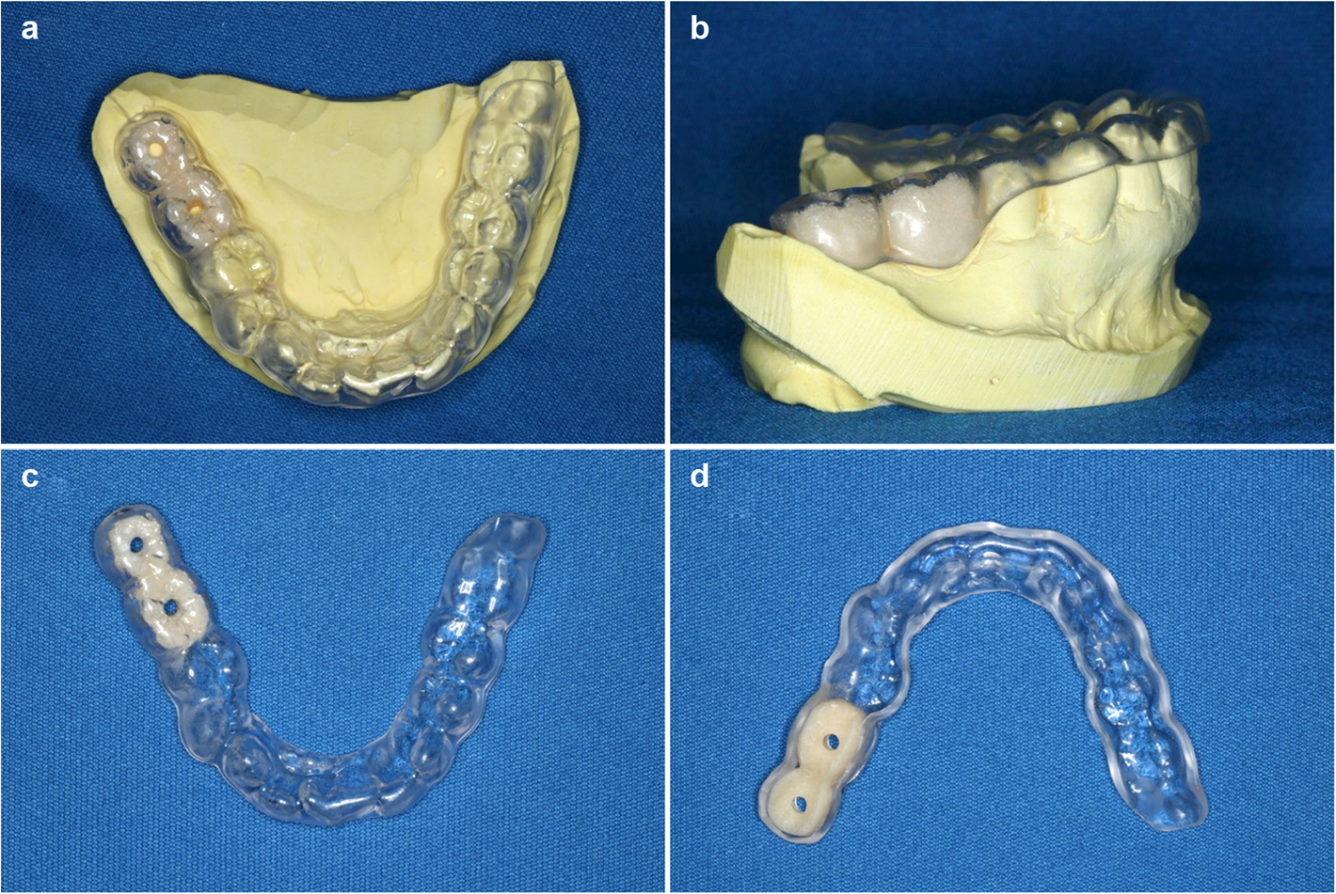
Diagnostic radiographic template. **a** Occlusal view with the diagnostic template mounted on the model. **b** Lateral view with the diagnostic template mounted on the model. **c** View of the occlusal surface of the diagnostic template. **d** View of the mucosal surface of the diagnostic template

**Fig. 2:**
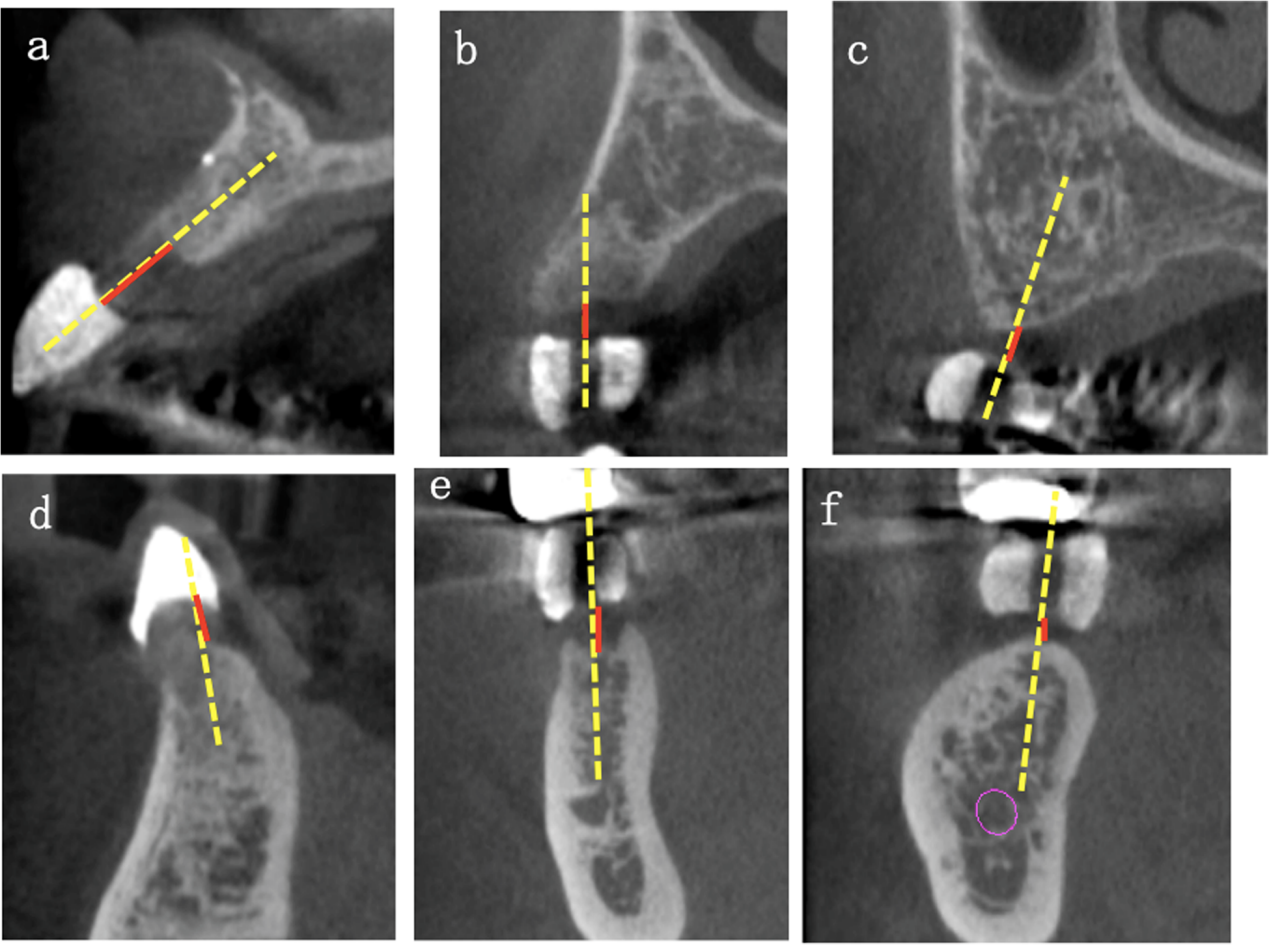
Cross-sectional image. The yellow dotted line indicates the ideal direction of implant insertion, as determined based on the radiographic stent, and set as the long axis. Red lines indicate the mucosal thickness as measured from the base of artificial teeth of the diagnostic template to the surface of the alveolar bone. **a** Maxillary anterior region. **b** Maxillary premolar region. **c** Maxillary molar region. **d** Mandibular anterior region. **e** Mandibular premolar region. **f** Mandibular molar region

Individual radiographic templates were fabricated for each patient before CBCT was performed. Each template contained artificial teeth, made of radiopaque resin composite, in the edentulous area to indicate the position and shape of the future implant crown. Each artificial tooth had a hole, 2 mm in diameter, penetrating the center of the artificial tooth perpendicular to the occlusal plane to indicate the center of the planned implant crown (Fig. 1). CBCT scans were obtained using the 3DX system (Morita, Tokyo) and exported in the digital imaging and communication in medicine (DICOM) format. The data were imported into the software used for planning implant surgery (coDiagnostiX, Dental Wings, Montreal, Canada). On a cross-sectional image of the central hole of the artificial tooth, the ideal direction of implant insertion was determined based on the diagnostic template and set as the long axis. Thereafter, the vertical mucosal thickness was determined as the vertical distance, on the crest, between the base of the artificial tooth and the surface of the alveolar bone (Fig. 2). All measurements were performed by the same oral and maxillofacial radiologist of over 20 years’ experience. The measurements were repeated three times and the average value was recorded as the mucosal thickness.

The study was performed according to the guidelines of the Declaration of Helsinki and approval was obtained from the Ethical Committee of Kanagawa Dental University (Approval No. 276).

### Evaluation and statistical analyses

The analyses were reviewed by an independent statistician. Using the mucosal thickness data obtained from the CT images, comparative analyses were performed between the maxilla and mandible, according to sex, location of edentulous site in the maxilla and mandible, and percentage of sites with a vertical mucosal thickness of ≤2 mm.

The Mann-Whitney *U* test was used for comparisons according to jaw, sex, and thickness greater or lesser than 2 mm, while Bonferroni’s multiple comparison tests were used for comparisons among the edentulous sites in each jaw. All statistical analyses were performed using the Statistical Package for the Social Sciences (SPSS) version 21 (International Business Machines [IBM], Japan, Ltd., Tokyo, Japan), with *p* values less than 0.05 considered significant.

## Results

A total of 125 eligible patients (53 men and 72 women; mean age: 57.0 years) and 296 sites were included in this study. Evaluated sites included 41 in the maxillary anterior region, 45 in the maxillary premolar region, 36 in the maxillary posterior region, 14 in the mandibular anterior region, 45 in the mandibular premolar region, and 115 in the mandibular posterior regions.

**Fig. 3:**
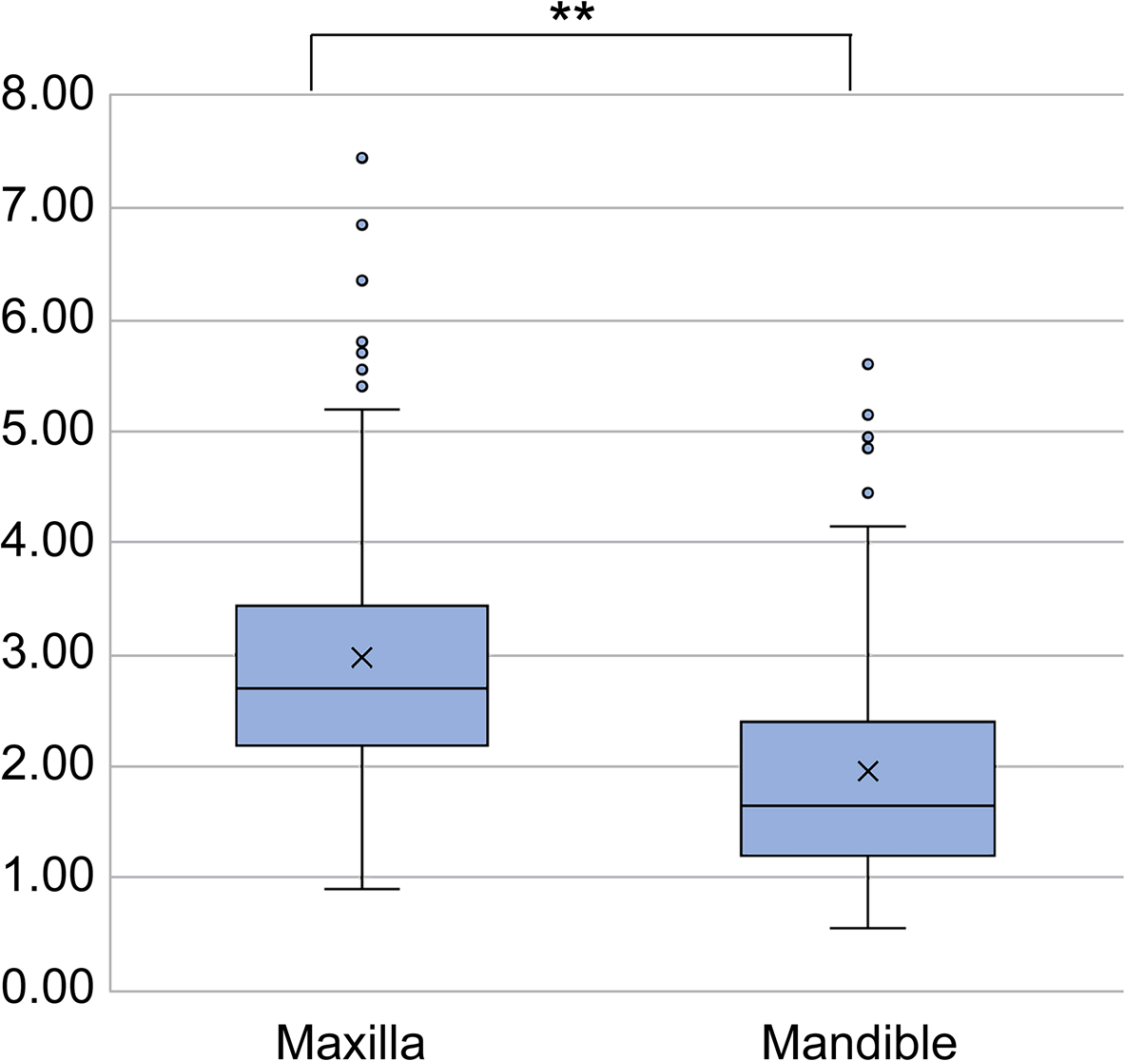
Mean MT values in the maxilla and mandible. The mean maxillary MT value was significantly greater than the mean mandibular MT value (*p*<0.001)

**Fig. 4:**
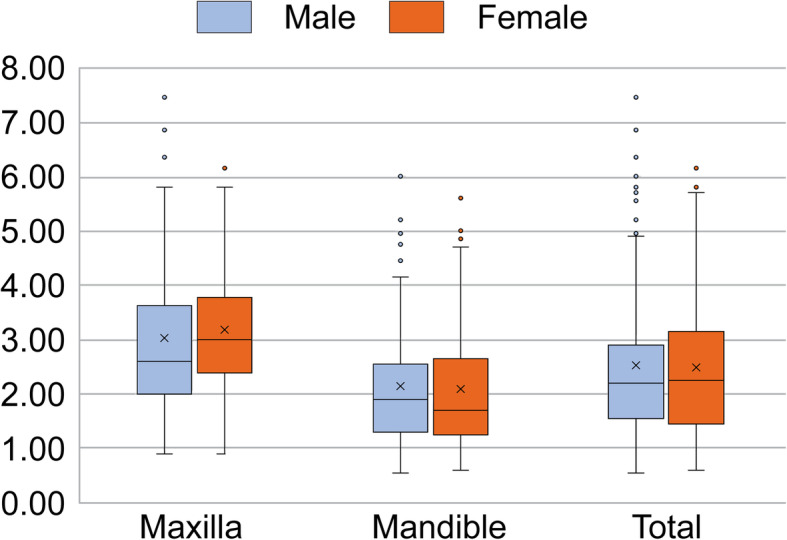
Comparison of the mean mucosal thickness (MT) of edentulous sites according to jaw and sex

The mean mucosal thickness was 3.0 ± 1.3 mm and 2.0 ± 1.0 mm in the maxilla and mandible, respectively, and the difference was statistically significant (*p* <0.001) (Fig. 3). The mean mucosal thickness was 2.5 ± 1.4 mm and 2.5 ± 1.2 mm in men and women, respectively, and the difference was not significant. In the maxilla, the mean mucosal thickness was 3.0 ± 1.6 mm and 3.2 ± 1.1 mm in men and women, respectively, while in the mandible, it was 2.1 ± 1.2 mm and 2.1 ± 1.2 mm in men and women, respectively. Again, no significant differences were found (Fig. 4).

(3) Table 1 shows the location and sex distribution of edentulous regions in which the mucosal thickness was measured.

**Table 1:** Location and sex distribution of edentulous regions studied

Edentulous region	Male	Female	Total
Maxilla			
Incisors and canines	24	17	41
Premolars	21	24	45
Molars	13	23	36
Mandible			
Incisors and canines	6	8	14
Premolars	19	26	45
Molars	53	62	115
Total	136	160	296

**Fig. 5:**
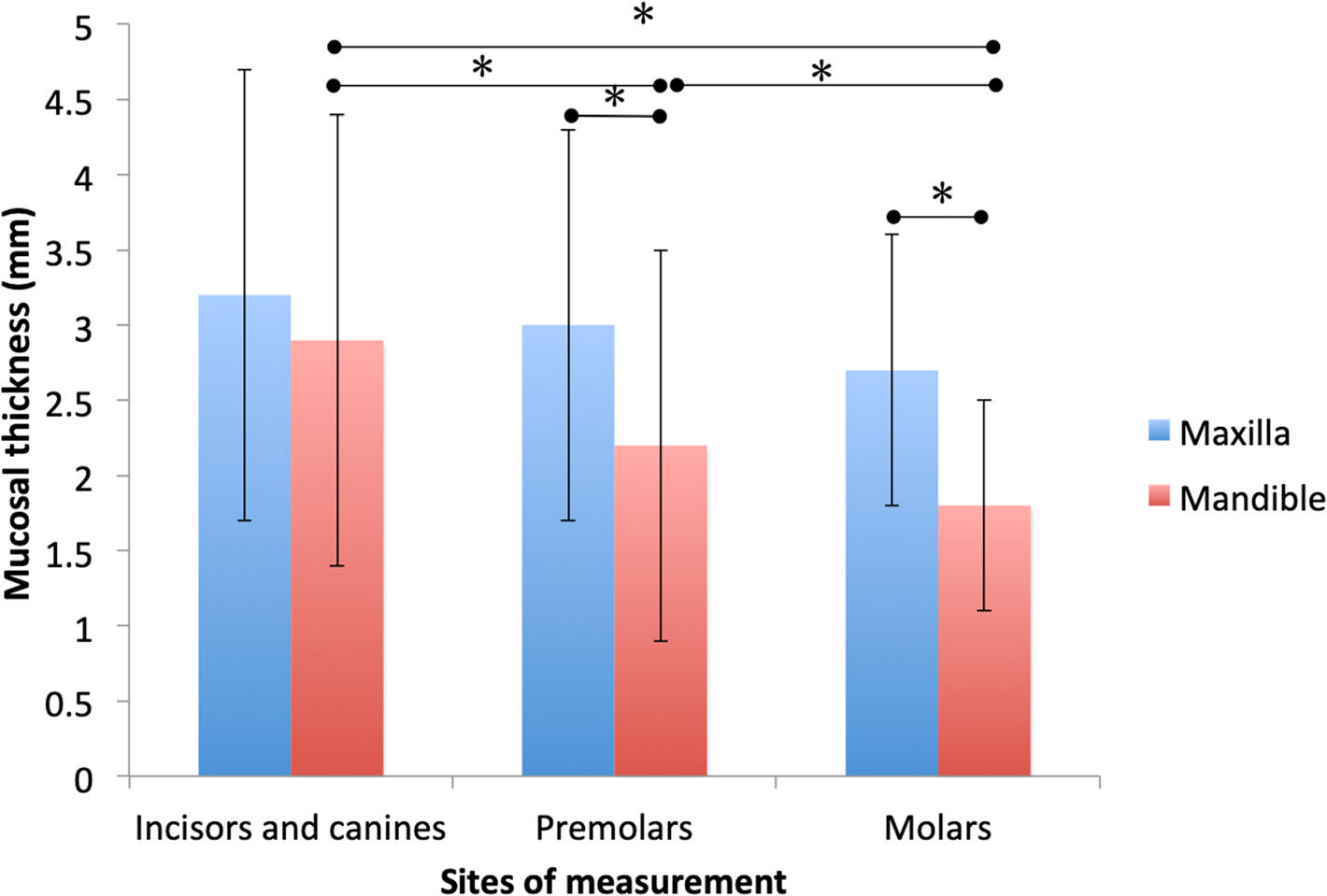
Comparison of mucosal thickness between the maxilla and mandible at each site. *p<0.05

In the maxilla, the mean mucosal thickness in the anterior region (3.2 ± 1.5 mm) was the greatest, followed by that in the premolar (3.0 ± 1.3 mm) and molar (2.7 ± 0.9 mm) regions, with no significant differences among them. Similarly, in the mandible, the mean mucosal thickness in the anterior region (2.8 ± 1.3 mm) was the greatest, followed by that in the premolar (2.3 ± 1.3 mm) and molar (1.8 ± 0.7 mm) regions. In contrast with the maxilla, significant differences were found in the mandible between the anterior and premolar regions, the anterior and molar regions, and the premolar and molar regions (Fig. 5). Interestingly, in both the maxilla and mandible, the mucosa was the thickest in the anterior region, followed by the premolar and molar regions. Additionally, in the premolar and molar regions, the mucosal thickness in the maxilla was significantly greater than that in the mandible.

In the maxilla, a mucosal thickness of ≤2 mm was found in 18% of total sites and in 17.1%, 20.0%, and 16.7% of the anterior, premolar, and molar regions, respectively. However, in the mandible 63.5% of total sites had a mucosal thickness of ≤2 mm, with 41.7%, 56.8%, and 66.1% in the anterior, premolar, and molar regions, respectively. Therefore, in the mandibular premolar and molar regions, more than half of the sites had a mucosal thickness of 2 mm or less, and even in the esthetically demanding maxillary anterior regions, approximately 20% of the sites showed a mucosal thickness of less than 2 mm (Table 2).

**Table 2:** Ratio of sites with mucosal thickness (MT) less than or equal to 2 mm

MT of 2 mm or less	Incisors and canines	Premolars	Molars	Total
Maxilla	17.1%	20.0%	16.7%	18.0%
Mandible	41.7%	56.8%	66.1%	63.5%

## Discussion

This study shows several tendencies regarding the variations in mucosal thickness. Edentulous alveolar mucosa in the maxilla is thicker than that of the mandible. In both the maxilla and the mandible, the edentulous alveolar mucosa was thickest in the anterior region, followed by the premolar and molar region. Accordingly, the edentulous alveolar mucosa should be thinnest in the mandibular posterior sites. The present study shows that 60% of the mucosal thickness of mandibular posterior sites was less than 2 mm.

In previous studies, the mucosal thickness was measured using anesthetic needles, files, periodontal probes, and a raised flap. These methods involved invasive procedures. Ultrasound equipment is another option to measure mucosal thickness. However, it has some drawbacks, such as operational difficulties and limited applicability [6, 14]. Lau et al. [15] measured gingival thickness in pig mandibles using CBCT. A high correlation coefficient of 0.995 was obtained on comparing the data with direct measurement results, indicating the accuracy of CBCT measurements. Ueno et al. [16] evaluated the mucosal thickness of a human cadaver by spiral CT and concluded that the measurement was precise except for very thin mucosa (<1 mm). Based on the results reported by Lau et al. [15], Ueno et al. [16], and those of the present study, a diagnostic template and CBCT might be useful for obtaining information on both soft and hard tissues via a single CT acquisition.

The well-known success criteria for dental implants are the speed and amount of crestal bone loss after the placement of superstructures [17]. Many factors contributing to crestal bone loss have been discussed, such as surgical trauma, implant positioning, design, and diameter, smoking, microgaps, abutment height, and biological width [18–20].

Studies have found a strong relationship between mucosal thickness around implants and crestal bone loss. In particular, some researchers have indicated that peri-implant mucosa thinner than 2 mm had a significantly greater amount of crestal bone loss around the dental implant compared to mucosa thicker than 2 mm both in the postoperative and post-prosthetic periods [2, 21, 22]. Vervaeke et al. [2] investigated how the probing pocket depth (PPD) and bone level around implants with different abutment heights changed over time; they demonstrated increased bone loss and PPD with decreased abutment height and suggested that the preoperative mucosal thickness had a major influence on the peri-implant biological width. In this study, the mean mucosal thickness in the mandibular premolar and molar regions was less than 2 mm, indicating that the mucosal condition is likely to cause bone loss during implant treatment. Linkevicius et al. [23] and Vervaeke et al. [2] also obtained similar results in their studies with platform-switched implants. Additional consideration should be given to the biological width before selecting the implant system and determining the depth of insertion in cases with a preoperative mucosal thickness of 2 mm or less [4]. We hope to further investigate the influence of mucosal thickness on bone volume and thereby develop a pre-implantation diagnostic protocol.

Biological width and preoperative thickness of the edentulous alveolar mucosa seem to substantially affect the peri-implant tissue and implant restoration in esthetically demanding regions by determining bone resorption, PPD, and susceptibility to peri-implantitis.

The planning of implant treatment can be performed more appropriately with information on mucosal thickness. Specifically, when planning implant treatment for the mandibular molar region, the practitioner can explain the risk associated with thin mucosa to the patient before taking his/her CBCT. In addition, it may be necessary to differentiate cases according to the mucosal thickness and establish an optimal implant placement protocol in each situation. For example, as Linkevicius suggested [24], a case with thin mucosa might need a soft tissue graft on top of the crest or crestal bone reduction at the time of implant placement.

The major limitation of the present study is the convenience sampling due to its retrospective nature, which potentially encompasses selection bias and uncontrolled intervening variables. Considering the fact that samples were drawn from a particular population, there may be biases due to ethnicity, regionality, and dominant age group of the population. The average age of the subjects in this study was 57 years; therefore, care should be taken when applying the findings to young patients. However, patients receiving implant treatment are generally the elderly irrespective of regional and ethnic dissimilarity; therefore, generation bias is not necessarily a concern. To offset the potential bias of convenient sampling, we collected a relatively large sample, including 125 partially edentulous patients with 296 sites.

Another limitation is the difference in the number of evaluated sites in each region. The mandibular molar region was predominant (115 sites), while in the mandibular anterior region only 14 sites were evaluated. This inconsistency may have distorted the statistical analysis.

In this study, the mucosal thickness was evaluated, only once, before implant surgery. No investigations have been conducted on the remodeling of the peri-implant bone and the accompanying change in the thickness of the peri-implant mucosa that may occur after implant placement and prosthesis installation. The postoperative changes in the implant site and mucosal thickness should pursue in further research. However, currently, it is considered that the measurement of mucosal thickness by CBCT as carried out in the present study is difficult due to implant artifacts. Therefore, it is necessary to use an auxiliary measurement method. Furthermore, the present study was an analysis of frequency distribution, and the fundamental tendency of distributions and associated clinical implications might not be overturned. Therefore, practitioners should note that the detailed ratios reported in this study might vary depending on clinical circumstances. In future studies, clinical parameters such as age, ethnicity, duration after extraction, variations in tooth position, and gingival biotype, can be controlled to eliminate any potential biases.

## Conclusions

We measured the mucosal thickness using a diagnostic template and CBCT and, within the limitations of the study, found that mean mucosal thickness in the maxilla is greater than in the mandible. There seems to be no gender difference in mucosal thickness. The mucosa in anterior region is the thickest, followed by the premolar and molar regions, both in the maxilla and mandible. The majority of the sites in the mandibular molar region had a mucosal thickness of less than 2 mm. Practitioners might be able to develop an optimal dental implant treatment plan for long-term biologic and esthetic stability by considering these factors.

## Data Availability

The data sets of the current study are available from the corresponding author upon reasonable request.

## Abbreviations

CBCT: Cone-beam computed tomography
CAD/CAM: Computer-aided design/computer-aided manufacturing
DICOM: Digital imaging and communication in medicine
IBM: International Business Machines
PPD: Probing pocket depth
